# Corneal dendritic cells and the subbasal nerve plexus following neurotoxic treatment with oxaliplatin or paclitaxel

**DOI:** 10.1038/s41598-021-02439-0

**Published:** 2021-11-24

**Authors:** Jeremy Chung Bo Chiang, David Goldstein, Azadeh Tavakoli, Terry Trinh, Jacob Klisser, Craig R. Lewis, Michael Friedlander, Thomas J. Naduvilath, Kimberley Au, Susanna B. Park, Arun V. Krishnan, Maria Markoulli

**Affiliations:** 1 School of Optometry and Vision Science, University of New South Wales, Sydney, Australia; 2 Department of Medical Oncology, Prince of Wales Hospital, Sydney, Australia; 3 Prince of Wales Clinical School, University of New South Wales, Sydney, Australia; 4 Brien Holden Vision Institute, Sydney, NSW Australia; 5 Brain and Mind Centre, Faculty of Medicine and Health, University of Sydney, Sydney, Australia

**Keywords:** Corneal diseases, Dendritic cells, Peripheral nervous system

## Abstract

Immune cell infiltration has been implicated in neurotoxic chemotherapy for cancer treatment. However, our understanding of immune processes is still incomplete and current methods of observing immune cells are time consuming or invasive. Corneal dendritic cells are potent antigen-presenting cells and can be imaged with in-vivo corneal confocal microscopy. Corneal dendritic cell densities and nerve parameters in patients treated with neurotoxic chemotherapy were investigated. Patients treated for cancer with oxaliplatin (n = 39) or paclitaxel (n = 48), 3 to 24 months prior to assessment were recruited along with 40 healthy controls. Immature (ImDC), mature (MDC) and total dendritic cell densities (TotalDC), and corneal nerve parameters were analyzed from in-vivo corneal confocal microscopy images. ImDC was increased in the oxaliplatin group (Median, Md = 22.7 cells/mm^2^) compared to healthy controls (Md = 10.1 cells/mm^2^, p = 0.001), but not in the paclitaxel group (Md = 10.6 cells/mm^2^). ImDC was also associated with higher oxaliplatin cumulative dose (r = 0.33, p = 0.04) and treatment cycles (r = 0.40, p = 0.01). There was no significant difference in MDC between the three groups (p > 0.05). Corneal nerve parameters were reduced in both oxaliplatin and paclitaxel groups compared to healthy controls (p < 0.05). There is evidence of elevation of corneal ImDC in oxaliplatin-treated patients. Further investigation is required to explore this potential link through longitudinal studies and animal or laboratory-based immunohistochemical research.

## Introduction

Platinum-based and taxane chemotherapeutic drugs are widely used in cancer treatment and are associated with potential peripheral neurotoxicity^1^. Chemotherapy-induced peripheral neuropathy is associated with debilitating and potentially chronic symptoms including numbness, paresthesia and compromised function, impacting up to 30% of cancer survivors^2^, with consequent adverse impact on quality of life and socioeconomic burden^3,4^. While the mechanisms underlying neurotoxicity are not fully understood, both axonal degenerative and neuroinflammatory processes have been implicated^1,5^. Studies of oxaliplatin and paclitaxel, two of the most commonly prescribed cytotoxic agents associated with peripheral neuropathy, have raised the possibility of the role of systemic inflammation in contributing to neurotoxicity^5,6^. Immune cell infiltration, particularly in the dorsal root ganglion, has also been investigated in their role in neurotoxicity^5,7–9^, although most studies are centered around animal models with limited research in humans^10^. In-vivo methods of observing and monitoring immune cell behavior is also lacking.

The cornea is a unique model for studying neural and immune changes because of its clarity and accessibility for in-vivo imaging. Corneal confocal microscopy is a powerful instrument used in clinical research to observe microscopic changes in patients with both ocular and systemic pathologies particularly peripheral neuropathic conditions^11,12^. It is capable of non-invasive high-resolution imaging of small nerve fibers and resident immune cells, namely dendritic cells, in the subbasal nerve plexus of the cornea. This is highly advantageous over other established methods of small nerve and immune cell imaging, including visualization of intraepidermal nerve fiber densities and Langerhans cells from invasive skin biopsies^13^. It is also less time consuming and has been increasingly used to observe neuroimmune features in the cornea including corneal dendritic cell density^14^ and behavior^15^. Dendritic cells are the most potent antigen-presenting cells in the body and are the primary responders responsible for initiating inflammatory and immune responses to damage or toxicity. They are the main resident immune cells in the cornea, and can be differentiated morphologically into immature and mature cell types^16^. Larger presence of immature dendritic cells is usually associated with an enhanced ability to mount an inflammatory response, whereas mature dendritic cells are derived from immature dendritic cells overexpressing costimulatory molecules such as CD80/CD86 and major histocompatibility complex (MHC) II to enhance their ability to active immunological processes^17^. Unlike immature dendritic cells, mature cells suggest the presence of active inflammation^18^.

Studies have shown an increase in the total number of corneal dendritic cells in other axonal degenerative conditions including diabetic peripheral neuropathy^19,20^, and mature corneal dendritic cells have also been shown to be associated with increased systemic inflammatory signaling involving receptors of tumor necrosis factors (TNF)^19^. Notably, ocular surface changes such as immune cell infiltration or tear film inflammatory mediators often reflect the status of systemic inflammation^21^. While evidence of corneal nerve fiber loss has been shown with neurotoxic chemotherapy in both human and animal models^22,23^, the changes in resident dendritic cells with chemotherapy have been limited to isolated case reports^24^ and the potential changes in treated patients across different major drug types remain unknown. The current study is novel in its investigation of in-vivo corneal dendritic cell densities and corneal nerve parameters in patients treated with oxaliplatin or paclitaxel. The second aim was to correlate these corneal neuroimmune features with the severity of peripheral neuropathy.

## Methods

### Study design and patient selection

This cross-sectional study was approved by the South Eastern Sydney Health Service Human Research Ethics Committee. Participants provided written informed consent after explanation of the nature and possible consequences of the study in accordance with the tenets of the Declaration of Helsinki. All methods were conducted in accordance with relevant guidelines and regulations.

Eighty-seven patients who had completed neurotoxic chemotherapy 3 to 24 months prior to assessment (oxaliplatin, n = 39; paclitaxel, n = 48) were consecutively recruited by convenience sampling from the Department of Medical Oncology, Prince of Wales Hospital (Sydney, Australia). A group of age-matched healthy controls (n = 40) were recruited for comparison. Sample size was assessed based on power calculations undertaken using G^*^Power 3.1.9.4 (Heinrich Heine University, Dusseldorf, Germany). As there is currently no quantitative data in neurotoxic chemotherapy, this was based on the corneal dendritic cell density in diabetic participants (17.73 ± 1.45 cells/mm^2^) compared to controls (6.94 ± 1.58 cells/mm^2^)^20^. A minimum sample of 20 participants in each group was estimated to have 80% power to detect a minimum mean difference of 1.7 ± 1.6 cells/mm^2^ at the 5% level of significance after accounting for Bonferroni corrected multiple comparisons.

Patients were excluded if they had a history of other medical conditions known to cause peripheral neuropathy including diabetes and chronic kidney disease, chronic demyelinating polyneuropathy, Sjogren’s syndrome and Charcot-Marie-Tooth disease. Medical records were also checked and the patients questioned during assessment for any such conditions including excessive alcohol consumption^25^. Pregnant and lactating women, patients with a history of ocular trauma, ocular surgery or refractive surgery, active ocular conditions including iritis, corneal edema, herpetic ulcers, corneal dystrophies or glaucoma requiring treatment with intraocular pressure lowering eye drop medication were excluded. Those with significant corneal epitheliopathy indicated by grade > 2 fluorescein staining on the Efron scale^26^, habitual soft and rigid contact lens wear, receiving corticosteroid eyedrops or systemic corticosteroid treatment, hormonal agents or oral retinoids at the time of assessment or those with known allergies to anesthetic eyedrops were also excluded.

### Clinical ocular surface assessments

Visual acuity assessment and slit lamp biomicroscopy were conducted on all participants. Symptomatology of ocular surface dysfunction was assessed with the Ocular Surface Disease Index (OSDI), a validated 12-item questionnaire developed to assess the severity of dry eye disease in relation to three subcategories: severity of ocular symptoms, visual function and environmental triggers^27^. The total score ranges from 0 to 100, with a total score > 12 indicating potential presence of dry eye disease^28^. Corneal integrity and staining according to the Efron scale^26^ was assessed by instilling a fluorescein strip moistened with saline solution (sodium chloride 0.9%). Tear film break up time (TBUT) was also determined by assessing the time between a blink and first signs of break in the fluorescein pattern. Corneal staining and TBUT were part of the dry eye assessment as markers of tear film homeostasis^29^.

### In-vivo corneal confocal microscopy

In-vivo corneal confocal microscopy was conducted according to previously published methods^22^. Briefly, a laser scanning confocal microscope (Heidelberg Retinal Tomograph III with Rostock Corneal module; Heidelberg Engineering GmbH, Heidelberg, Germany) was performed on participants after the cornea was anaesthetized with sterile 0.4% benoxinate hydrochloride (oxybuprocaine hydrochloride). Five to eight images best representing the central cornea with less than 20% overlap between images^30^, and three to five images of the inferior whorl region were identified. A validated automated image analysis software (ACCMetrics, The University of Manchester Intellectual Property UMIP, Manchester, United Kingdom)^31,32^ was used to analyze corneal nerve fiber length (CNFL, mm/mm^2^), corneal nerve fiber density (CNFD, no/mm^2^) and inferior whorl length (IWL, mm/mm^2^). The average nerve fiber length (ANFL = $\frac{CNFL+IWL}{2}$) was also calculated.

**Figure 1:**
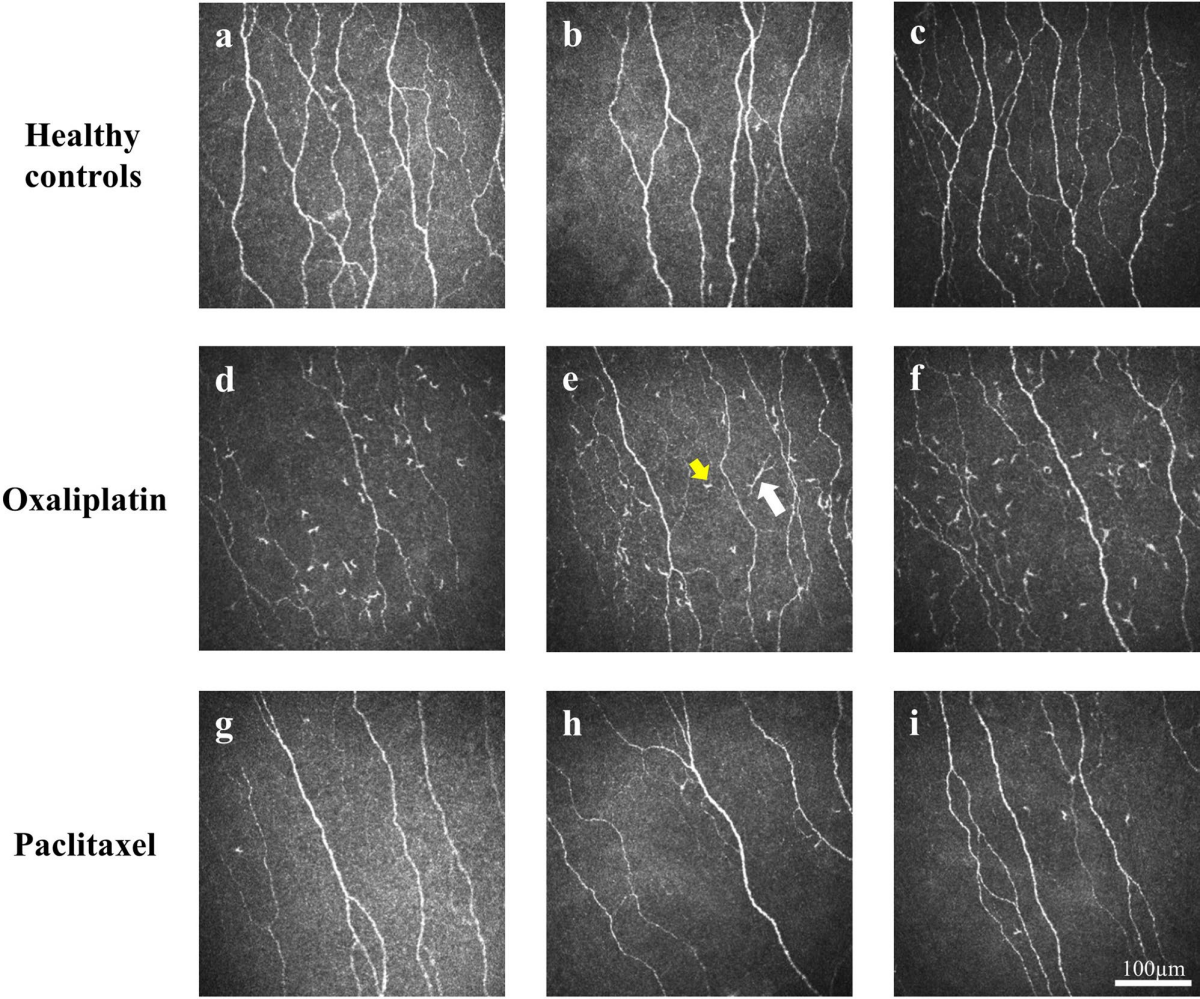
Representative in-vivo corneal confocal microscopy images. Images shown are in healthy controls (**a–c**), oxaliplatin-treated (**d–f**) and paclitaxel-treated (**g–i**) patients. Immature dendritic cells (ImDC, yellow arrow in **e**) and mature dendritic cells (MDC, white arrow in **e**) are depicted.

Corneal dendritic cells identified in the corneal epithelium subbasal layer were defined as reflective bodies with dendritiform processes^16^ and were manually quantified by a masked, independent observer from the in-vivo corneal confocal microscopy images of the central cornea (Fig. 1). These were further classified into immature and mature dendritic cells. Immature dendritic cells (ImDC) were defined as small, reflective cell bodies with short discernible dendrites, tapered ends or processes (< 25 µm)^16,19^. These cells are able to undergo maturation to increase their potency in antigen processing and presentation leading to an inflammatory or immune response^33^. Mature dendritic cells (MDC) were defined as reflective, slender cell bodies often with multiple long dendritiform processes extending out from the main cell body (> 25 µm)^16,19^. These are usually regarded as markers of overt and active inflammation^34^. The total dendritic cell densities including ImDC and MDC (TotalDC) were also noted.

### Peripheral neurotoxicity assessments

Assessment of peripheral nerve health have been described previously^22^. Neuropathy status in all participants was graded using the reduced version of the Total Neuropathy Scale (TNSr©Johns Hopkins University) which assesses clinical parameters across 8 domains (sensory symptoms, weakness symptoms, pin prick sensibility, vibration threshold, strength, deep tendon reflexes, sensory nerve, and motor nerve conduction studies) with grades ranging from 0 to 4 where 0 signifies no deficit and 4 represents severe changes^35,36^. The total score ranges from 0 to 32. Nerve conduction studies were undertaken in the sural sensory and tibial motor nerves as per standard protocols^37^. Clinical assessment was also conducted using the National Cancer Institute Common Terminology Criteria for Adverse Events (NCI-CTCAE) version 3 sensory subscale for sensory peripheral neuropathy^38^. The European Organization for Research and Treatment of Cancer Quality of Life—Chemotherapy induced Peripheral Neuropathy questionnaire (EORTC QLQ-CIPN20) is a 20-item validated questionnaire and was used as a patient reported outcome assessing peripheral neuropathy symptomatology and potential impact on functional activities of daily living^39^. Each participant indicated the degree to which they have experienced each item by rating it from 1 to 4, where 1 signifies ‘not at all’ and 4 represents ‘very much’. The total scores were then linearly converted to a 0–100 scale, with higher scores indicating higher symptom burden^39^.

### Statistical analysis

Statistical analysis was conducted using SPSS (version 25.0, IBM Corp., Armonk; NY, USA). As a preliminary assessment of the reproducibility of dendritic cell density measures, the intraclass correlation coefficient (ICC) based on absolute agreement (two-way random effects, average measures) was 0.994 (95% confidence interval (CI) 0.988–0.996) for immature dendritic cells, and 0.988 (95% CI 0.980–0.993) for mature dendritic cells between two masked observers (JK, AT). ICC based on absolute agreement for intra-reliability (two-way mixed effects, single measures) was 0.984 (95% CI 0.947–0.994) for ImDC, and 0.966 (95% CI 0.917–0.987) for MDC.

Normality was assessed with Shapiro–Wilk test and Q-Q plots. Preliminary data checks of dendritic cell densities indicated that there was large variability relative to the mean, with the distribution being skewed to the right. Hence a logarithmic transformation was applied to the raw dendritic cell density data. Univariate analysis using one-way ANOVA for parametric data (age and body mass index, BMI) and Kruskal Wallis test for non-parametric data (clinical ocular and peripheral neuropathy measures) with post-hoc Bonferroni adjustment of p-values was used to compare measures between the three groups. The proportion of participants in each group with dry eye disease (OSDI > 12 with presence of corneal staining, with grade ≥ 1 on the Efron scale or TBUT < 10 s) was also determined^29^. Chi squared test of independence was used for comparing gender proportions and the prevalence rates of dry eye disease between participants.

A general linear model was conducted to assess if dendritic cell densities (ImDC, MDC, TotalDC) and corneal nerve parameters (CNFD, CNFL, IWL, ANFL) were significantly different between the three groups of participants. Each of the 7 outcome variables were analyzed as a separate model with the ability to detect group differences after accounting for possible confounding factors. A confounder was a patient characteristic such as age, gender, BMI and dry eye diagnosis that were significantly different between study groups. Partial correlation analysis was conducted to assess the correlation of dendritic cell densities with corneal nerve parameters after adjusting for age. The correlation between dendritic cell densities with neurotoxic drug parameters including cumulative dose and number of treatment cycles, as well as neuropathy severity, clinician- and patient-reported outcomes were also assessed for each treatment group.

For the drug group showing significant differences in dendritic cell densities, differences between patients were further stratified according to higher and lower neuropathy severity groups based on the median of the TNSr scores (≤ Median TNSr: lower neuropathy severity; > Median TNSr: higher neuropathy severity) were investigated with independent t-test (age and BMI), Chi-squared test of independence (gender and dry eye proportions) or Mann–Whitney U test (clinical ocular and peripheral neuropathy measures). A general linear model was also used to assess the differences in dendritic cell densities and corneal nerve parameters in patients according to higher and lower neuropathy severity while accounting for confounding factors as described before. Post hoc pairwise comparisons between groups were adjusted using Bonferroni correction. Two-tailed level of significance was set at p < 0.05.

## Results

### Clinical and demographic data

The clinical and demographic data for patients treated with oxaliplatin and paclitaxel are presented in Table 1. Peripheral neuropathy measures including TNSr scores, sural and tibial nerve amplitudes, NCI-CTCAE and EORTC QLQ-CIPN20 scores are also presented in Table 1. There were no significant differences in age between the three groups (p = 0.50). The paclitaxel group had higher BMI than healthy controls (p = 0.02). There were more females in the paclitaxel group compared to the oxaliplatin group (χ = 27.31, df = 1, p < 0.001) and healthy controls (χ = 18.94, df = 1, p < 0.001) as paclitaxel is primarily used for treating breast and gynecological cancers. Sural nerve amplitudes were significantly reduced in the oxaliplatin and paclitaxel groups (p < 0.001) compared to healthy controls, while tibial nerve amplitudes were not significantly different between groups. Treatment regimens and systemic comorbidities are noted in Supplementary Table S1.

**Table 1:** Demographic, ocular surface measures, corneal nerve and neuropathy measures of patients who received oxaliplatin or paclitaxel for cancer treatment.

Characteristics	Participants		
	Oxaliplatin (n = 39)	Paclitaxel (n = 48)	Healthy controls (n = 40)
Age	60.8 ± 9.5	58.6 ± 10.4	58.9 ± 7.9
BMI, kg/m^2^	27.0 ± 5.0	27.4 ± 5.4^†^	24.4 ± 4.1
Gender (female)	18 (46%)	46 (96%)^‡,¶^	23 (58%)
**Primary cancer site**			
Colorectal	32 (82%)	0 (0%)	N/A
Pancreatic	4 (10%)	0 (0%)	
Other gastrointestinal	3 (8%)	0 (0%)	
Breast	0 (0%)	41 (85%)	
Gynecological	0 (0%)	7 (15%)	
**UICC staging prior to treatment**			
I	1 (3%)	10 (21%)	N/A
II	6 (15%)	16 (33%)	
III	17 (44%)	15 (31%)	
IV	12 (31%)	0 (0%)	
Unspecified	3 (8%)	7 (15%)	
Number of treatment cycles	8 [5.8–10.3]	10.5 [7–12]	N/A
Mean cumulative dose, mg/m^2^	663.78 ± 230.30	852.21 ± 165.28	N/A
Period post-treatment, months	10.3 ± 7.3	13.5 ± 7.3	N/A
NCI-CTCAE for peripheral sensory neuropathy (range: 0–4)	2 [1, 2]	1 [1, 2]	N/A
EORTC QLQ-CIPN20 (range: 0–100)	19.3 [12.3–31.6]	15.8 [5.7–29.4]	N/A
TNSr scores (range: 0–32)	5 [3–11]^‡^	3.5 [1–7]^‡^	0 [0–1]
Sural nerve amplitude, µV	6.6 [3.3–11.7]^§^	12.8 [9.0–17.0]^‡^	18.5 [9.3–26.3]
Tibial nerve amplitude, mV	9.8 [3.9–14.1]	12.6 [7.2–16.6]	12.5 [8.9–14.8]
logVA	0.14 [0.10–0.30]	0.14 [0.02–0.22]	0.10 [0–0.18]
OSDI	0 [0–6.25]	8.33 [0–18.23]*,**	0 [0–4.17]
TBUT	11 [6.8–13]	10 [7–12]	12 [10–12.5]
Corneal staining	0 [0–0]	0 [0–1]	0 [0–2]

Data is presented as mean ± standard deviation or median [interquartile range Q1–Q3]. Compared with healthy controls: ^†^p = 0.02; ^‡^p < 0.001; ^§^p = 0.002; **p = 0.001; ***p = 0.007; ****p = 0.003; *****p = 0.006. Compared with oxaliplatin group: ^¶^p < 0.001; *p = 0.008.

*BMI* body mass index, *NCI-CTCAE* National Cancer Institute Common Terminology Criteria for Adverse Events, *EORTC QLQ-CIPN20* the European Organization for Research and Treatment of Cancer Quality of Life—Chemotherapy-induced Peripheral Neuropathy questionnaire, *TNSr* reduced version of Total Neuropathy Scale, *logVA* log visual acuities, *OSDI* Ocular Surface Discomfort Index, *TBUT* tear film break up time, *CNFD* corneal nerve fiber density, *CNFL* corneal nerve fiber length, *IWL* inferior whorl length, *ANFL* average nerve fiber length.

Visual acuity was similar across the oxaliplatin, paclitaxel and healthy control groups (p = 0.10, Table 1). The paclitaxel group had worse OSDI scores compared with the oxaliplatin group (p = 0.008) and healthy controls (p = 0.001). The prevalence of dry eye disease in the paclitaxel was 13/48 (27%) compared to 2/39 (5%) in the oxaliplatin group (χ = 7.27, df = 1, p = 0.007), and 1/40 (3%) in the healthy controls (χ = 9.86, df = 1, p < 0.001).

### Dendritic cell densities and corneal nerves with oxaliplatin and paclitaxel treatment

**Figure 2:**
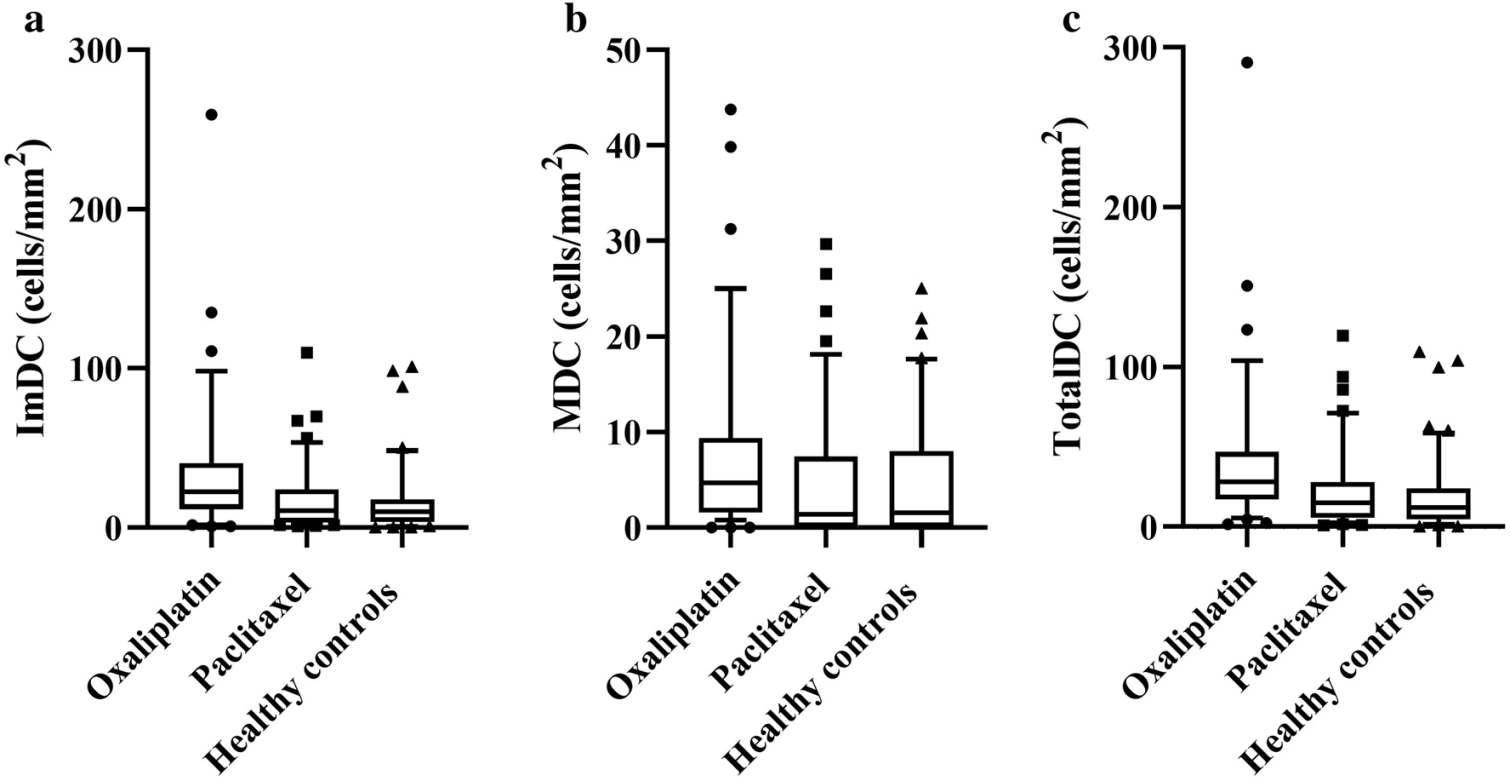
Dendritic cell density comparisons. Boxplots and whiskers representing the 10th to 90th percentile of (**a**) immature dendritic cell density (ImDC), (**b**) mature dendritic cell density (MDC), and (**c**) total dendritic cell density (TotalDC) in patients treated with paclitaxel or oxaliplatin and healthy controls.

Representative images of the corneal nerves and dendritic cells for the oxaliplatin, paclitaxel and healthy control groups are shown in Fig. 1. Table 2 shows the model based estimates for dendritic cell densities and corneal nerve parameters for the three groups following adjustment for age, gender, BMI and dry eye diagnosis. Figure 2 shows the distribution of dendritic cell densities for each group.

**Table 2:** Model based estimates from general linear model after adjusting for age, body mass index, gender and dry eye diagnosis for dendritic cell densities and corneal nerve parameters in participants.

Parameters	Participants					
	Oxaliplatin (n = 39)		Paclitaxel (n = 48)		Healthy controls (n = 40)	
	Mean	95% CI	Mean	95% CI	Mean	95% CI
ImDC, cells/mm^2^	18.2 (1.28)^†^	10.9 to 30.1	11.2 (1.09)	7.0 to 17.6	8.5 (0.98)	4.8 to 14.3
MDC, cells/mm^2^	3.9 (0.69)	2.2 to 6.6	2.7 (0.57)	1.5 to 4.5	2.3 (0.52)	1.1 to 4.1
TotalDC, cells/mm^2^	24.2 (1.40)^‡^	15.0 to 38.8	15.8 (1.23)	10.2 to 24.2	11.2 (1.09)	6.7 to 18.3
CNFD, no/mm^2^	22.4^¶^	19.7 to 25.1	21.7^§^	19.4 to 24.1	26.9	24.2 to 29.6
CNFL, mm/mm^2^	13.4*	12.0 to 14.7	13.3**	12.1 to 14.5	15.8	14.5 to 17.2
IWL, mm/mm^2^	12.2***	10.6 to 13.9	13.3	11.8 to 14.8	15.6	13.9 to 17.3
ANFL, mm/mm^2^	12.9***	11.5 to 14.2	13.3****	12.1 to 14.4	15.7	14.3 to 17.1

Data is presented as mean from model based estimates and log mean value in parentheses, with 95% confidence intervals (CI). Compared with healthy controls: ^†^p = 0.03; ^‡^p = 0.02; ^¶^p = 0.01; ^§^p = 0.005; *p = 0.004; **p = 0.006; ***p = 0.001; ****p = 0.008.

*ImDC* immature dendritic cell density, *MDC* mature dendritic cell density, *TotalDC* total dendritic cell density, *CNFD* corneal nerve fiber density, *CNFL* corneal nerve fiber length, *IWL* inferior whorl length, *ANFL* average nerve fiber length.

Based on the general linear model, ImDC was significantly higher in the oxaliplatin group (Median [Interquartile range]: 22.7 cells/mm^2^ [11.7–40.6]) compared to healthy controls (10.1 cells/mm^2^ [3.5–17.6], p = 0.03). However, there was no significant difference in ImDC between healthy controls and the paclitaxel group (10.6 cells/mm^2^ [3.3–24.0], p = 0.99). A similar increase in TotalDC (28.1 cells/mm^2^ [17.2–46.9]) was observed in the oxaliplatin group compared to healthy controls (12.2 cells/mm^2^ [4.7–27.9], p = 0.02). There were no significant differences in ImDC and TotalDC between the oxaliplatin group and the paclitaxel group (ImDC: 10.6 cells/mm^2^ [3.3–24.0], p = 0.32; TotalDC: 14.8 cells/mm^2^ [5.7–28.1], p = 0.36). MDC was not significantly different between the three groups (p > 0.05; Table 2).

Both CNFD and CNFL were reduced in the oxaliplatin (CNFD: 23.2 ± 6.3 no/mm^2^, p = 0.01; CNFL: 13.7 ± 2.9 mm/mm^2^, p = 0.004) and paclitaxel groups (CNFD: 21.7 ± 7.6 mm/mm^2^, p = 0.005; CNFL: 13.1 ± 3.8 mm/mm^2^, p = 0.006) compared to healthy controls (CNFD: 28.0 ± 6.4 no/mm^2^; CNFL: 16.3 ± 3.2 mm/mm^2^). IWL and ANFL were similarly reduced in the oxaliplatin group (IWL: 12.5 ± 3.7 mm/mm^2^, p = 0.001; ANFL: 13.2 ± 2.8 mm/mm^2^, p = 0.001) compared to healthy controls (IWL: 16.3 ± 3.9 mm/mm^2^; ANFL: 16.3 ± 3.1 mm/mm^2^). ANFL was also reduced in the paclitaxel group (13.4 ± 3.8 mm/mm^2^, p = 0.008) compared to healthy controls. There were no significant differences in corneal nerve parameters between the oxaliplatin and paclitaxel groups (p > 0.05; Table 2).

**Figure 3:**
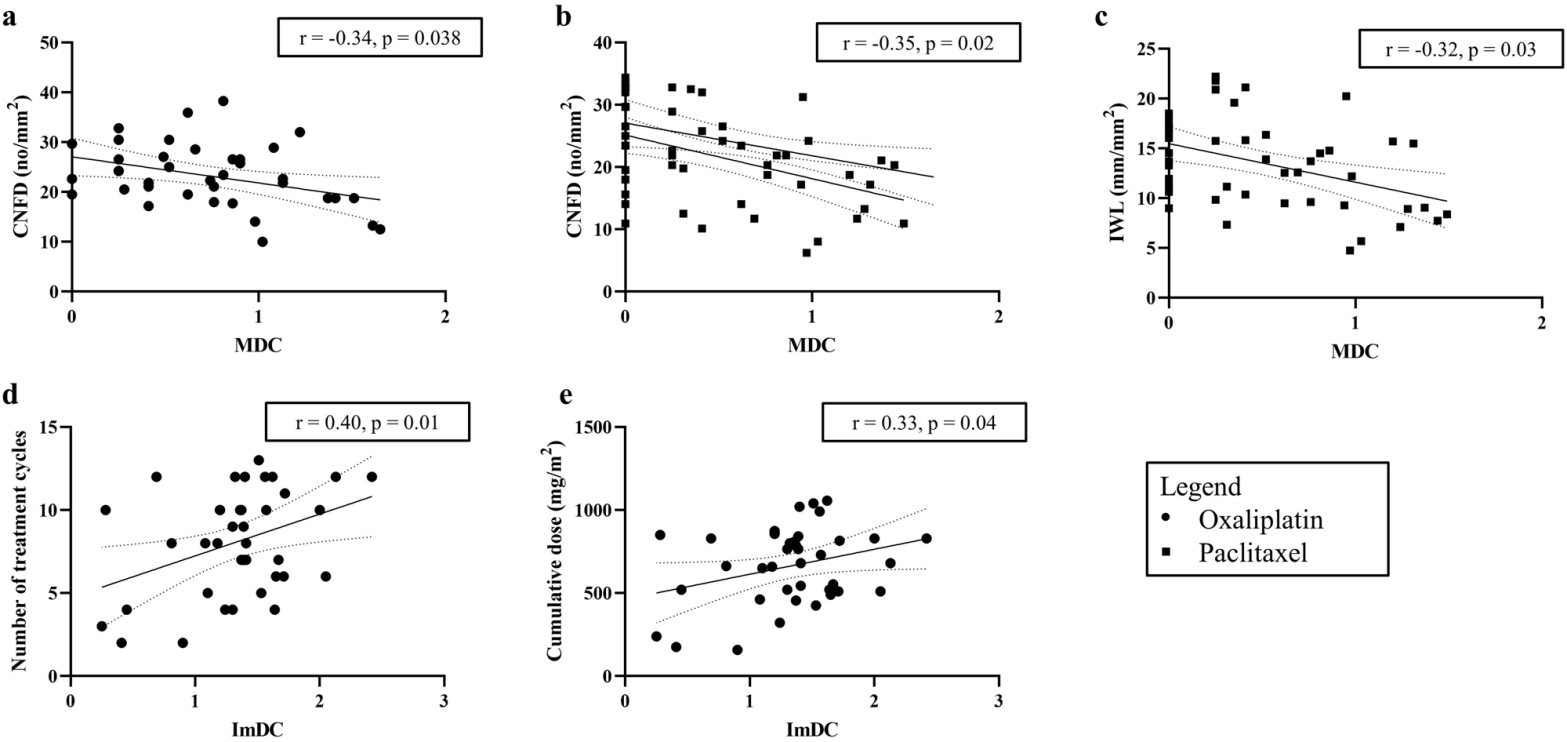
Dendritic cell density correlations. Scatterplots with linear regression and 95% confidence interval bands showing association between log values of higher mature dendritic cell density (MDC) and loss of corneal nerve fiber density (CNFD) in (**a**) oxaliplatin-treated patients and (**b**) paclitaxel-treated patients. Correlation between MDC and loss of inferior whorl length (IWL) in paclitaxel-treated patients is shown in (**c**). Increased immature dendritic cell density (ImDC) in oxaliplatin-treated patients was associated with (**d**) higher numbers of treatment cycles associated and (**e**) higher cumulative dose of oxaliplatin.

Correlation analysis showed that higher MDC was associated with CNFD loss in the oxaliplatin group (r = 0.34, p = 0.038; Fig. 3a, Table 3). Higher MDC were also associated with loss of CNFD and IWL in paclitaxel (r = 0.35, p = 0.02 and r = 0.32, p = 0.03; Fig. 3b,c; Table 3). Conversely, there was an association between higher MDC with higher CNFD (r = 0.32, p = 0.048) and higher CNFL (r = 0.34, p = 0.04) in healthy controls. Higher number of treatment cycles in the oxaliplatin group was associated with the increase in ImDC (r = 0.33, p = 0.04; Fig. 3d) and TotalDC (r = 0.37, p = 0.02) observed in the oxaliplatin group (Supplementary Table S2). Similarly, higher cumulative dose of oxaliplatin was associated with elevated ImDC (r = 0.40, p = 0.01; Fig. 3e) and TotalDC (r = 0.42, p = 0.009). There were no associations between dendritic cell densities with treatment data or neuropathy severity in the paclitaxel group (Supplementary Table S3). Additionally, no associations were found between corneal nerve parameters with treatment data or neuropathy severity in the oxaliplatin or paclitaxel groups (Supplementary Table S2 and Supplementary Table S3).

**Table 3:** Associations between dendritic cell densities and corneal nerve parameters for patients treated with oxaliplatin or paclitaxel.

	CNFD	CNFL	IWL
**Oxaliplatin**			
ImDC	−0.15 (p = 0.39)	−0.05 (p = 0.79)	−0.09 (p = 0.59)
MDC	−**0.34 (p = 0.038)**	−0.10 (p = 0.56)	−0.16 (p = 0.36)
**Paclitaxel**			
ImDC	0.08 (p = 0.58)	0.15 (p = 0.33)	−0.01 (p = 0.93)
MDC	−**0.35 (p = 0.02)**	−0.17 (p = 0.28)	−**0.32 (p = 0.03)**

Data is reported as r (p-value) with statistically significant correlations highlighted in bold.

*ImDC* immature dendritic cell density, *MDC* mature dendritic cell density, *TotalDC* total dendritic cell density, *CNFD* corneal nerve fiber density, *CNFL* corneal nerve fiber length, *IWL* inferior whorl length, *ANFL* average nerve fiber length.

### Corneal neuroimmune features in peripheral neuropathy

The oxaliplatin group, which was the only treated group that showed a significant increase in ImDC and TotalDC compared to healthy controls, were stratified according to lower neuropathy severity group (TNSr ≤ 5), and higher neuropathy severity group (TNSr > 5; Table 4). Of the 39 oxaliplatin-treated participants, 20 had peripheral neuropathy in the higher severity (51%). Demographics, treatment data, clinical ocular and neuropathy measures are presented in Supplementary Table S4. The number of treatment cycles and mean cumulative oxaliplatin dose received was greater in the higher neuropathy severity group compared to the lower neuropathy severity group (p = 0.02 and p = 0.006). There were no patients with dry eye disease in the lower neuropathy severity group (0%), while the high neuropathy severity group had 2/19 patients with dry eye disease (11%), although the difference was not statistically significant (χ = 2.22, df = 1, p = 0.14).

**Table 4:** Model based estimates from general linear model for corneal neuroimmune features in oxaliplatin-treated patients post-cessation stratified according to lower (reduced version total neuropathy scale (TNSr) ≤ 5) and higher neuropathy severity (TNSr > 5) with healthy controls.

Parameters	Oxaliplatin-treated participants			
	Higher neuropathy severity (n = 19)		Lower neuropathy severity (n = 20)	
	Mean	95% CI	Mean	95% CI
ImDC, cells/mm^2^	45.8 (1.67)	6.8 to 280.8	44.7 (1.66)	5.6 to 315.2
MDC, cells/mm^2^	3.3 (0.63)	0.3 to 12.8	1.6 (0.41)	0 to 8.1
TotalDC, cells/mm^2^	49.8 (1.71)	9.5 to 244.5	39.4 (1.61)	6.4 to 218.8
CNFD, no/mm^2^	20.0	10.7 to 29.4	16.7	6.6 to 26.8
CNFL, mm/mm^2^	11.3	6.7 to 15.8	9.3	4.4 to 14.2
IWL, mm/mm^2^	11.4	5.2 to 17.6	12.8	9.5 to 16.1
ANFL, mm/mm^2^	12.0	7.8 to 16.2	11.0	6.4 to 15.5

Data is presented as mean ± standard deviation or median [interquartile range Q1–Q3]. For the dendritic cell densities and corneal nerve parameters, data is presented as mean from model based estimates with 95% confidence intervals (CI). The log mean values for dendritic cell densities are presented in parentheses.

*CNFD* corneal nerve fiber density, *CNFL* corneal nerve fiber length, *IWL* inferior whorl length, *ANFL* average nerve fiber length, *ImDC* immature dendritic cell density, *MDC* mature dendritic cell density, *TotalDC* total dendritic cell density.

There was no statistically significant difference in corneal nerve parameters between the higher neuropathy severity group (CNFD: 24.2 ± 6.8 no/mm^2^ ; CNFL: 14.5 ± 2.7 mm/mm^2^; IWL: 12.1 ± 3.1 mm/mm^2^; ANFL: 13.3 ± 2.3 mm/mm^2^) compared to the lower neuropathy severity group (CNFD: 22.3 ± 5.9 no/mm^2^, p = 0.18; CNFL: 13.0 ± 3.1 mm/mm^2^, p = 0.10; IWL: 13.0 ± 4.3 mm/mm^2^, p = 0.69; ANFL: 13.1 ± 3.3 mm/mm^2^, p = 0.35). Dendritic cell densities were also similar between the higher neuropathy severity group (ImDC: 22.7 cells/mm^2^ [5.5–46.1]; MDC: 4.7 cells/mm^2^ [2.3–15.6]; TotalDC: 30.5 cells/mm^2^ [17.2–56.3]) compared to the lower neuropathy severity group (ImDC: 27.0 cells/mm^2^ [12.9–35.2], p = 0.96; MDC: 2.7 cells/mm^2^ [0.8–6.8], p = 0.11; TotalDC: 25.9 cells/mm^2^ [17.4–41.4], p = 0.58).

## Discussion

Our study investigated corneal neuroimmune features observed with in-vivo corneal confocal microscopy in patients who have completed treatment with neurotoxic chemotherapy with either paclitaxel or oxaliplatin. A major focus was to assess the potential effect of neurotoxic chemotherapy on corneal dendritic cells which are pertinent resident immune cells. A significant increase in ImDC in the oxaliplatin group was observed well after treatment cessation, even after accounting for age, gender, BMI and presence of dry eye disease. However, this was not associated with neuropathy severity. Conversely, there was no elevation in density observed in paclitaxel-treated patients. Dry eye disease, which has been associated with inflammatory changes on the ocular surface causing symptoms of discomfort and surface damage^40^, is unlikely to contribute to the differences as there was no evidence of significant ocular surface dysfunction in the oxaliplatin group. This further suggests that the increase in ImDC in the oxaliplatin group is indicative of low-level inflammatory presence, rather than an active clinical or pathological manifestation such as that seen in severe keratitis or keratopathies, which would have been indicated by significant increases in MDC^41,42^. ImDC elevation is associated with an enhanced ability to phagocytose antigens and mount an inflammatory response and hence may indicate a sustained, chronic low-level inflammation in oxaliplatin-treated patients as opposed to an active overt inflammation^43^.

In the current study, the increase in ImDC in the oxaliplatin group was associated with prolonged prior exposure to oxaliplatin indicated by the number of treatment cycles and cumulative dose of oxaliplatin. Oxaliplatin has been shown to increase inflammatory mediators including tumor necrosis factor alpha (TNF-α) and interleukin 1 beta (IL-1β)^44^, and immune cells such as dendritic cells and enhanced cytotoxic T cells elsewhere in lung carcinoma tissues of murine models^45^. While systemic inflammatory mediators of treated patients were not investigated in the current study, a previous study of oxaliplatin-treated patients has shown an increase in pro-inflammatory mediators including the transcriptional factor nuclear factor-kappa light chain enhancer of B cells (NF-κB) measured immediately after treatment cessation^46^. These findings may be of clinical importance in developing more targeted anti-inflammatory or immunosuppressive approaches while minimizing adverse reactions associated with traditional steroidal agents^47^. Whether these elevated levels are sustained chronically post-treatment is yet to be investigated. However, platinum compounds including oxaliplatin and cisplatin can persist within the body including the skin and blood years after completion of treatment^48,49^, suggesting that the observed increase in ImDC may be indicative of the presence of low levels of residual platinum exerting a chronic but subclinical impact on potent antigen-presenting cells.

The current study also showed no significant changes in dendritic cells in paclitaxel-treated participants. Although paclitaxel also increases inflammatory cytokines including IL-1β and IL-6 in the dorsal root ganglia^50^, it has been shown to have little impact on antigen presentation capabilities of dendritic cells^51^, inducing a reduction in dendritic cell size and loss of dendritiform processes^52^. In contrast, oxaliplatin seems to have a more stimulatory impact on dendritic cells^45,53^, which may explain the increase in densities in oxaliplatin-treated patients but not in paclitaxel-treated patients. We also identified higher prevalence of dry eye disease in the paclitaxel group compared to the oxaliplatin group and healthy controls. This may be due to the greater proportion of females in the paclitaxel group as female gender has been associated with higher risk of dry eye disease^54^, although other unexplored factors may play a role. Dry eye disease is an ocular surface condition where cellular disruption occurs due to the loss of homeostasis regulating tear film quality and ocular surface health which leads to symptomatic burden^40^. Further research is required to investigate whether paclitaxel could additionally disrupt ocular surface homeostasis through corneal nerve dysfunction or neuropeptide dysregulation such as expression of substance P involved in inflammatory processes as has been shown previously^55^, thus contributing to dry eye disease.

The association between MDC and corneal nerve loss in the drug groups observed in the current study may reflect a neuroimmune interaction between these immune cells and corneal nerves as reflected by other studies in both human and animal models^56–58^. Conversely, in healthy individuals, higher MDC seems to be associated with larger corneal nerve parameters particularly higher CNFD and CNFL in our study. This positive association between has been observed previously; whereby dendritic cells support corneal nerve growth and maintenance in healthy humans and animals^14,59^. This may occur through expression of neurotrophic factors such as ciliary neurotrophic factors (CNTF) in the healthy state^60^. However, higher MDC in pathological conditions such as diabetes or exposure to toxins may be linked with corneal nerve loss and dysfunction through prolonged adverse pro-inflammatory effects^19^. This supports a potential differential role of MDC in regulating corneal nerve morphology depending upon the degree of activation of the immune system and the state of disease or health.

Our study did not find an association between severity of the neuropathy in either drug group and dendritic cell densities, which may reflect the complex interplay of more pertinent factors which could potentially contribute to persistent chronic neuropathy including downstream inflammatory processes involving cytokine and chemokine action^46^ and genetic polymorphisms^61,62^. In addition, there remains a lack of research on systemic inflammation in cancer survivors and its effect on dendritic cell populations^63–66^. The current study is further limited by its cross-sectional nature, and longitudinal studies are required to measure corneal neuroimmune changes in patients during and after treatment. Any potential longstanding impact of the cancer itself on corneal nerves, dendritic cells and peripheral nerves which was not assessed in the current study should also be further investigated. However, paraneoplastic syndromes are usually subacute or fulminant and subside after anticancer treatment^67^. Ferdousi et al. showed a reduction in corneal nerves in patients with gastrointestinal cancer before treatment, and a subsequent increase with three cycles of platinum-compound treatment thought to be due to nerve regeneration^68^. Larger studies with longer follow up periods are required to confirm such observations including the relative impact of neurotoxic drugs on corneal nerves. Laboratory-based immunohistochemical studies would also be important to further understand corneal dendritic cell function and behavior in response to oxaliplatin and paclitaxel. Markers of systemic inflammation were not investigated in the current study which focused on the observation of potential corneal dendritic cell changes with in-vivo corneal confocal microscopy. Further studies to better understand the potential link between systemic and local ocular inflammatory markers in neurotoxicity are required.

The current study revealed an elevation of corneal ImDC in patients who have received oxaliplatin treatment, but not paclitaxel, although this was not associated with peripheral neuropathy severity. Further investigation is required to explore this potential link through longitudinal studies and animal or laboratory-based immunohistochemical research. Importantly, loss of corneal nerves was also prominent in treated patients. Future studies should explore these corneal neural and inflammatory changes detectable in an in-vivo, non-invasive manner during neurotoxic chemotherapy treatment from baseline.

## Supplementary Information

Supplementary Table S1.

Supplementary Table S2.

Supplementary Table S3.

Supplementary Table S4.

## Data Availability

The datasets generated and analyzed during the study are available from the corresponding author on reasonable request.
