# Supplementary material for: Corneal dendritic cells and the subbasal nerve plexus following neurotoxic treatment with oxaliplatin or paclitaxel
Source: Sci Rep. 2021 Nov 24;11:22884. doi: 10.1038/s41598-021-02439-0 (PMC8613280; doi:10.1038/s41598-021-02439-0)
Supplement: Supplementary file 1 — Supplementary Table S1. [file 41598_2021_2439_MOESM1_ESM.docx]

**Supplementary Table S1. Treatment regimen of oxaliplatin and paclitaxel, and systemic comorbidities of participants.** Treatment regimen details: FOLFOX (fluorouracil, leucovorin, oxaliplatin); FOLFOXIRI / FOLFIRINOX (fluorouracil, leucovorin, oxaliplatin, irinotecan); CAPOX (capecitabine, oxaliplatin); AC (doxorubicin, cyclophosphamide).

|  | **Oxaliplatin (n = 39)** | **Paclitaxel (n = 48)** |
| --- | --- | --- |
| Treatment regimen  Oxaliplatin  FOLFOX  FOLFOXIRI / FOLFIRINOX  CAPOX  Paclitaxel  Paclitaxel weekly  AC+Paclitaxel weekly/2-weekly  Paclitaxel weekly/3-weekly+Carboplatin | 28 (72%)  7 (18%)  4 (10%) | 23 (48%)  14 (29%)  11 (23%) |
| Systemic comorbidities  Hypertension  Hypercholesterolemia  Osteoarthritis  Hyperthyroidism/Hypothyroidism  Anemia  Hyperlipidemia  Migraine  Non-alcoholic fatty liver disease  Sleep apnea  Coronary heart disease  Gout  Hyperparathyroidism  Vitamin D deficiency  Hypokalemia  Hypomagnesemia | 11 (28%)  5 (13%)  4 (10%)  2 (5%)  2 (5%)  1 (3%)  1 (3%)  1 (3%)  1 (3%)  1 (3%)  2 (5%)  0 (0%)  0 (0%)  1 (3%)  1 (3%) | 7 (15%)  2 (4%)  5 (11%)  2 (4%)  2 (4%)  1 (2%)  1 (2%)  1 (2%)  1 (2%)  2 (4%)  0 (0%)  1 (2%)  1 (2%)  0 (0%)  0 (0%) |
