# Supplementary material for: Corneal dendritic cells and the subbasal nerve plexus following neurotoxic treatment with oxaliplatin or paclitaxel
Source: Sci Rep. 2021 Nov 24;11:22884. doi: 10.1038/s41598-021-02439-0 (PMC8613280; doi:10.1038/s41598-021-02439-0)
Supplement: Supplementary file 2 — Supplementary Table S2. [file 41598_2021_2439_MOESM2_ESM.docx]

**Supplementary Table S2.** **Correlation matrix of the association between dendritic cell densities and corneal nerve parameters with treatment regimen data and neurophysiological measures for oxaliplatin-treated patients.** Data is reported as r (p-value). Abbreviations: ImDC, immature dendritic cell density; MDC, mature dendritic cell density; TotalDC, total dendritic cell density; CNFD, corneal nerve fiber density; CNFL, corneal nerve fiber length; IWL, inferior whorl length; TNSr, reduced version of Total Neuropathy Scale; NCI-CTCAE, National Cancer Institute Common Terminology Criteria for Adverse Events; EORTC QLQ-CIPN20, the European Organization for Research and Treatment of Cancer Quality of Life – Chemotherapy-induced Peripheral Neuropathy questionnaire.

|  | **Cumulative dose** | **Number of treatment cycles** | **TNSr** | **NCI-CTCAE** | **EORTC QLQ-CIPN20** |
| --- | --- | --- | --- | --- | --- |
| **ImDC** | **0.33 (p = 0.04)** | **0.40 (p = 0.01)** | 0.04 (p = 0.81) | 0.11 (p = 0.53) | 0.07 (p = 0.69) |
| **MDC** | 0.14 (p = 0.42) | 0.13 (p = 0.43) | 0.20 (p = 0.23) | 0.13 (p = 0.44) | -0.10 (p = 0.57) |
| **TotalDC** | **0.37 (p = 0.02)** | **0.42 (p = 0.009)** | 0.14 (p = 0.42) | 0.12 (p = 0.46) | 0.05 (p = 0.78) |
| **CNFD** | 0.11 (p = 0.53) | 0.17 (p = 0.32) | 0.13 (p = 0.44) | -0.10 (p = 0.57) | -0.01 (p = 0.97) |
| **CNFL** | 0.22 (p = 0.19) | 0.30 (p = 0.07) | 0.24 (p = 0.15) | -0.05 (p = 0.75) | 0.06 (p = 0.74) |
| **IWL** | 0.21 (p = 0.22) | 0.27 (p = 0.11) | -0.16 (p = 0.34) | 0.05 (p = 0.77) | -0.12 (p = 0.46) |
