# Supplementary material for: Corneal dendritic cells and the subbasal nerve plexus following neurotoxic treatment with oxaliplatin or paclitaxel
Source: Sci Rep. 2021 Nov 24;11:22884. doi: 10.1038/s41598-021-02439-0 (PMC8613280; doi:10.1038/s41598-021-02439-0)
Supplement: Supplementary file 3 — Supplementary Table S3. [file 41598_2021_2439_MOESM3_ESM.docx]

**Supplementary Table S3. Correlation matrix of the association between dendritic cell densities and corneal nerve parameters with treatment regimen data and neurophysiological measures for paclitaxel-treated patients.** Data is reported as r (p-value). Abbreviations: ImDC, immature dendritic cell density; MDC, mature dendritic cell density; TotalDC, total dendritic cell density; CNFD, corneal nerve fiber density; CNFL, corneal nerve fiber length; IWL, inferior whorl length; TNSr, reduced version of Total Neuropathy Scale; NCI-CTCAE, National Cancer Institute Common Terminology Criteria for Adverse Events; EORTC QLQ-CIPN20, the European Organization for Research and Treatment of Cancer Quality of Life – Chemotherapy-induced Peripheral Neuropathy questionnaire.

|  | **Cumulative dose** | **Number of treatment cycles** | **TNSr** | **NCI-CTCAE** | **EORTC QLQ-CIPN20** |
| --- | --- | --- | --- | --- | --- |
| **ImDC** | -0.31 (p = 0.06) | -0.16 (p = 0.30) | 0.13 (p = 0.39) | 0.12 (p = 0.44) | 0.08 (p = 0.59) |
| **MDC** | -0.20 (p = 0.23) | -0.29 (p = 0.05) | 0.02 (p = 0.90) | -0.08 (p = 0.61) | -0.08 (p = 0.60) |
| **TotalDC** | -0.31 (p = 0.06) | -0.25 (p = 0.11) | 0.06 (p = 0.68) | 0.08 (p = 0.58) | 0.02 (p = 0.90) |
| **CNFD** | -0.11 (p = 0.49) | -0.13 (p = 0.39) | -0.04 (p = 0.78) | -0.05 (p = 0.76) | 0.07 (p = 0.63) |
| **CNFL** | -0.02 (p = 0.88) | -0.16 (p = 0.28) | -0.11 (p = 0.46) | -0.14 (p = 0.35) | -0.02 (p = 0.91) |
| **IWL** | 0.14 (p = 0.41) | -0.27 (p = 0.08) | -0.22 (p = 0.13) | -0.14 (p = 0.34) | -0.08 (p = 0.60) |
