# Supplementary material for: Corneal dendritic cells and the subbasal nerve plexus following neurotoxic treatment with oxaliplatin or paclitaxel
Source: Sci Rep. 2021 Nov 24;11:22884. doi: 10.1038/s41598-021-02439-0 (PMC8613280; doi:10.1038/s41598-021-02439-0)
Supplement: Supplementary file 4 — Supplementary Table S4. [file 41598_2021_2439_MOESM4_ESM.docx]

**Supplementary Table S4.** **Clinical ocular and peripheral neuropathy measures in oxaliplatin-treated patients post-cessation stratified according to lower (reduced version Total Neuropathy Scale (TNSr ≤ 5) and higher neuropathy severity (TNSr > 5) with healthy controls.** Compared with lower neuropathy severity group: † p = 0.02; ‡ p = 0.006; ¶ p = 0.02; § p < 0.001. Abbreviations: BMI, body mass index; EORTC QLQ-CIPN20, the European Organization for Research and Treatment of Cancer Quality of Life – Chemotherapy-induced Peripheral Neuropathy questionnaire; TNSr, reduced version of Total Neuropathy Scale; logVA, log visual acuities; OSDI, Ocular Surface Discomfort Index; TBUT, tear film break up time.

| **Parameters** | **Participants** | |
| --- | --- | --- |
|  | **Higher neuropathy severity (n = 19)** | **Lower neuropathy severity (n = 20)** |
| Age | 62.7 ± 9.4 | 59.1 ± 9.4 |
| BMI, kg/m^2^ | 27.7 ± 5.2 | 26.3 ± 4.9 |
| Gender (Female) | 53 (74%) | 11 (73%) |
| Cancer  Colorectal  Pancreatic  Gastrointestinal | 15 (79%)  2 (11%)  1 (5%) | 16 (80%)  2 (10%)  2 (10%) |
| UICC staging prior to treatment  I  II  III  IV  Unspecified | 0 (0%)  3 (16%)  7 (37%)  6 (32%)  3 (16%) | 1 (5%)  3 (15%)  10 (50%)  6 (30%)  0 (0%) |
| Number of treatment cycles | 10 [7-12] † | 6.5 [4-10] |
| Mean cumulative dose, mg/m^2^ | 764.92 ± 178.37 ‡ | 572.76 ± 237.50 |
| Period post-treatment, months | 8.1 ± 5.5 | 12.3 ± 8.2 |
| EORTC QLQ-CIPN20 (Range: 0-100) | 28.1 [14.8-33.3] ¶ | 17.1 [11.0-21.1] |
| TNSr scores (Range: 0-32) | 5 [3-7] | 1 [1-1] |
| Sural nerve amplitude, µV | 3.3 [0-8.4] § | 10.8 [6.6-13.6] |
| Tibial nerve amplitude, mV | 9.3 [3.1-12.6] | 11.4 [5.1-16.0] |
| logVA | 0.18 [0.10-0.30] | 0.14 [0.10-0.30] |
| OSDI | 0 [0-10.4] | 0 [0-4.2] |
| TBUT, s | 10 [6-13] | 12 [7-13] |
| Corneal staining | 0 [0-0.5] | 0 [0-0] |
